# Supplementary material for: Are carbon emissions trading and green financial instruments synergistic? -Comprehensive quantitative research based on content analysis
Source: PLoS One. 2024 Mar 7;19(3):e0298601. doi: 10.1371/journal.pone.0298601 (PMC10919601; doi:10.1371/journal.pone.0298601)
Supplement: S1 Data — (ZIP) [file pone.0298601.s001.zip › Data/地级市Z_F工作报告.zip!/║■─╧/╓Ω╓▐╩╨/└┤╫╘╣1⁄2╓┌║┼í╛┬φ┐╦╩2╛▌═°í┐_2009.docx]

各位代表：

　　现在，我代表市人民政府向大会作工作报告，请予审查，并请市政协各位委员和其他列席同志提出意见。

　　一、攻坚克难，基本实现良好开局

　　2008年，是株洲发展史上很不寻常的一年。我们在市委的坚强领导下，紧紧围绕“保二争一、科学跨越”战略目标，认真履行政府职责，加快推进“一化三基”，圆满完成了各项工作任务。

　　实现GDP910亿元，增长13.5%；财政收入82.1亿元，增长21.5%，其中一般预算收入46.7亿元，增长24.3%；全社会固定资产投资388亿元，增长34.4%，其中工业投资200亿元，增长38.9%；城镇居民人均可支配收入16500元，农民人均纯收入5700元，分别增长14%、13%。

　　（一）抓机遇，经济活力明显增加

　　“两型格局”基本确立。核心区、协调发展区、示范区三级布局初步形成，市区和株洲县、醴陵市的部分区域进入全省核心区，面积达1863平方公里，炎陵、茶陵、攸县成为协调发展区，云龙、天易示范区进入全省5大示范区范畴。“打造示范区、建设新株洲”的工作主题全面树立，34.41平方公里农用地变性获得国家五部委批复，为株洲未来争得了发展空间。“两型社会”建设总体实施方案、5个专业规划和湘江风光带二期、旧城改造等规划编制完成。时代大道等一批标志性工程已经启动，清水塘循环经济实施方案获得国家批准。

　　各项改革深入推进。61家市属国有企业改革完成扫尾，8家建设和商贸企业、18家集体企业改革全面部署，煤气、自来水、排水等公用事业改革正式启动。在全省率先由政府支付农田灌溉水费,直接为农民减负2644万元。城乡公共服务均等化、土地使用权有偿流转、建设用地增减挂钩等试点和集体林权制度改革相继铺开。国库集中支付改革加快推进。投融资改革不断深化。国有资产监管得到加强。

　　对外开放继续扩大。切实转变招商方式，新批外商投资企业40家，实际到位外资3.2亿美元，利用市外境内资金137.4亿元。不断拓展外贸新格局，完成进出口总额11.5亿美元，其中出口7亿美元。大力扶持非公经济，实现增加值495亿元，增长14.5%，年销售收入过亿元的非公企业增加到70家。

　　需要特别报告的是，在国家出台扩大内需政策的第一时间，我们反应迅速，应对有力，成效明显。到去年底，衔接落实项目34个、资金5.1亿元，资金数量居全省各市州前列。

　　（二）抓发展，产业优势明显强化

　　一产业在巩固中提升。实现农业增加值110亿元，增长6%。粮食生产再创佳绩，播种面积389万亩，总产创历史新高，达191万吨。规模养殖取得突破，年出栏生猪万头以上养殖场16个。农业产业化步伐加快，新上5000万元以上项目3个，发展专业合作经济组织206家，流转土地98.3万亩，占农用地总面积的10.7%，农产品加工实现总产值113亿元，增长18.7%。唐人神100万头猪现代化屠宰流水线竣工投产。醴陵、攸县继续保持全省县域经济10强县。

　　二产业在优化中壮大。实现工业增加值433亿元，增长16.3%，占GDP的比重提高到47.6%，其中规模工业增加值340亿元，增长18%。实施特殊政策、特殊奖励和特殊服务，“5115”工程有效推进，15家企业完成增加值132亿元，占规模工业增加值的38.8%。支持企业技术改造，实施500万元以上项目711个，增加81个，完成投入170亿元，增长30%，北汽控股南方生产基地、多晶硅、电力机车扩改、中小航空发动机基地、中材和华新新型干法水泥、中铁轨道扩能、株冶循环经济一期、株化PVC扩改等项目顺利推进。坚持非均衡发展战略，园区平台进一步夯实，实现技工贸总收入503亿元，增长30%。大力发展高新技术产业，重新认定高新技术企业70家，争取国家、省级科技项目65项，其中国家863计划6项，完成专利申请1320件，第六次荣获全国科技进步先进城市称号。建筑业稳步发展。

　　三产业在整合中放活。实现增加值305亿元，增长13.5%。金融运行平稳，年末各项存款余额712.7亿元，贷款余额329.8亿元，比年初分别增加74.7亿元、36.8亿元。个人存款户均达到1.3万元。新入驻股份制银行3家，证券、保险、期货市场等均有新的发展。“万村千乡”市场工程继续推进，农产品市场准入制度开始推行，市场监管力度加大，物价基本稳定，城乡贸易繁荣有序，实现社会消费品零售总额310亿元，增长25.2%。房地产业规范发展，完成投资80亿元。旅游产业稳步壮大，云阳山通过国家4A级旅游区验收。

　　（三）抓环境，城乡面貌明显改观

　　城市品位大幅提升。围绕创建国家卫生城市、国家交通管理模范城市和全国文明城市，突出抓了四项工作：一是实施“五改工程”。改造小街小巷157条、人行道板11条，完成砼道路改沥青38条、“穿衣戴帽”工程31个、10千伏架空电力线路入地10条。二是改善城区路网。石宋大道一期、泰山西路、黄河南路、铁西南路等主次干道建设全面竣工，株洲大桥、石峰大桥维修顺利结束，天台路、长江南路、人民路、芦淞路、中心广场改造按时完成，田心立交、新塘东路、湘江防洪二期等工程有序启动，湘江五桥建设进度加快。同时，城区绿化、亮化有新的变化。三是加强城市管理。环卫作业市场化改革全面铺开，月度考核排名严格到位，城区主次干道实现全天候保洁，老旧住宅小区、城中村、城乡结合部、农贸市场和铁路沿线的综合整治初见成效，运行市场化、管理网格化、作业精细化、考核标准化的城市管理框架基本形成。四是优化交通秩序。智能交通监控系统启动建设，限摩、限货和禁鸣措施有效实施，标线标牌、公交站点和专用车道不断完善。随着这些工作的推进，城市更加清新、更具品位，创建国家卫生城市顺利通过国家暗检，有望一举成功。

　　农村条件大为改善。加快小城镇建设，完成投资11.6亿元，城镇化水平提高到49%。推进新农村建设“十百千”工程，整合项目340个，落实资金1.2亿元。投入5.1亿元，完成各类水利工程3.78万处，治理病险水库57座。解决了9.77万人的饮水不安全问题。加强农业综合开发，治理水土流失101.5平方公里，改造中低产田5.5万亩。完成工程造林15.7万亩。发放各类涉农补贴3.4亿元。农业综合机械化率达到52%。新建农村安居房2300栋。新建村级活动场所171个。在全省率先开通“12316”农民服务热线。

　　交通能源大步跨越。交通建设完成投资31亿元，创历史新高。长株高速、衡炎高速、武广客运专线顺利推进，炎睦高速、衡茶吉铁路开工建设。S321炎陵至睦村、S320茶陵至界化垄等3条干线公路基本建成。改造通乡公路339公里、通村公路1928公里。新建农村客运站15个。洮水水库建设有序推进，攸县煤电一体化项目顺利开工。投资4.4亿元，完成了1588个行政村的电网恢复重建和改造。新建沼气池9249个，总数突破7万个。

　　环境保护大力加强。深入开展“蓝天碧水净土”行动，顶住减少GDP20多亿元、税收2亿多元的巨大压力，果断关停和搬迁选矿药剂厂、化工助剂厂等污染企业和落后生产线113家（条），尤其是关闭电厂两台12.5万千瓦机组，爆破一座180米高的烟囱，在株洲环保史上写下了划时代的一笔。加快“一江两岸四港”治理，湘江株洲段水体质量保持国家Ⅲ类标准。完成了1737家服务单位煤改清洁能源工作，居民天然气用户达15.6万。大力开展城镇污水处理设施“三年全覆盖”行动，开工建设污水处理厂7个，其中龙泉二期已投入运行，城市污水处理率提高到66%。通过努力，城区空气良好天数达到340天，比上年增加24天，市区饮用水源水质达标率99.4%，主要污染物排放总量持续下降，砷、镉减排全面完成省政府下达的“十一五”任务。

　　（四）抓民生，社会建设明显进步

　　教育水平有效提升。教育强市全面启动，农村义务教育经费保障新机制全面落实，城乡义务教育学杂费全部免除，惠及学生29万人，资助贫困学生4.2万人。“普九”化债工作有序开展，新建农村合格学校50所。职业教育不断壮大，株洲职业技术学院、湖南铁路科技职业技术学院办学水平被教育部评估为优秀。教育创新力度加大，城区普通高中“小班化”教学经验在全省推广。

　　卫生体系不断健全。医疗基础设施进一步改善，市中心医院和中医院、中医伤科医院综合大楼如期开工；新改扩建乡镇卫生院22所，所有乡镇卫生院改扩建任务全部完成；新建社区卫生服务中心23个，城区社区卫生服务网络实现全覆盖。新型农村合作医疗制度全面推行，参合率达91.9%，发放补助1.6亿元，17.1万名农民受益。重大疾病防控机制不断健全，免费进行手足口病防治以及问题奶粉患儿的筛查和治疗，突发公共卫生事件得到有效处置。第四次荣获全国无偿献血先进城市。

　　保障力度持续加大。认真落实就业再就业政策，培训下岗失业人员1.8万人，新增城镇就业5.8万人，继续保持城镇“零就业家庭”动态为零，城镇登记失业率控制在4.5%以内；新增农村劳动力转移就业6.9万人。城镇职工基本养老、医疗、工伤、失业、生育保险覆盖面进一步提高，征缴力度进一步加大。城镇居民医疗保险全面实施。发放住房公积金个人贷款3.9亿元、廉租住房补贴1800万元，投资1.2亿元的首个廉租示范小区开工建设。新改扩建乡镇敬老院20所，实现每个乡镇拥有1所敬老院。城乡低保标准进一步提高，分别达到每月220元、45元。

　　其它事业繁荣有序。成功举办合唱节、读书月和炎帝陵公祭典礼。在全国率先建成100个乡村大舞台，免费开放博物馆、纪念馆等文化活动场所。市广电中心主体工程基本建成，农村广播电视“户户通”加快推进，1.6万人的“收视难”问题得到解决。圆满承办省第三届大众运动会，竞技体育实力不断增强。新档案馆建成启用。认真落实农村计生家庭奖励和扶助政策，符合政策生育率92.57%。募集慈善资金1.1亿元。投入9201万元，帮助6.5万人脱贫致富。妇女儿童、气象、市志、助残、老龄、民族宗教等工作有序开展。

　　社会管理得到加强。合泰、龙泉地区“三合一”整治取得阶段性成果。严格责任落实，实施“科技兴安”，未发生重特大安全事故。完善社会治安防控体系，深入开展严打整治，社会大局平稳可控。启动“法制株洲”建设，认真开展“大接访”活动。健全预案体系，应急能力进一步增强。积极开展和谐社区、文明社区创建和农村社区建设试点，深入推进村务公开，居（村）民自治水平明显提高。同时，全面落实了省、市实事任务。

　　这一年，政府自身建设同步加强。承办人大代表建议275件、政协委员提案317件，办结率和见面率均为100%，满意率99.3%。建成“数字株洲”指挥中心和市政府门户网站，所有县市区政府和市直机关实现网上公文传输。落实行政程序有关规定，工业投资项目行政审批代理制已经实施，限时办结制度开始建立。新闻发布工作规范推进，政务公开力度不断加大。加强公务员培训考核，队伍素质有效提高。加大审计监督和行政监察力度，行政效能和服务水平不断提升。同时，国防、民兵预备役、人防等工作扎实推进。

　　回顾2008年工作，我们深切地体会到，做好政府工作，必须以人为本，关注民生，人民群众的幸福感就是执政者的成就感，想群众所忧，急群众所盼，解群众所难，一切工作始于群众需求、终于群众满意；必须激情工作，规范管理，状态决定行动，制度至高无上，先行先试、敢闯敢试、边干边试，敢于担当、敢于负责、敢于批评，以奋发有为推动科学跨越，以科学规范提升管理水平；必须勤政廉政，依法行政，勤廉乃为政之本，行政必须依法，爱岗敬业、勤勉为民，认认真真做事，清清白白做人，依法依规履行职责，光明磊落行使权力。

　　各位代表！2008年，我们经历了风雨，见证了彩虹；付出了艰辛，赢得了发展。面对年初的罕见冰灾、下半年的全球金融危机，我们取得的成绩十分来之不易。这是市委正确领导、市人大支持监督的结果，是市政协和各民主党派、工商联、无党派人士、各人民团体参政议政的结果，是全市人民团结拼搏、艰苦奋斗的结果。在此，我代表市人民政府，向全体人大代表、政协委员，向驻株部队官兵和全市人民，向关心支持株洲发展的社会各界，致以崇高的敬意！

　　同时，我们也清醒地认识到前进中的困难和问题。主要是：发展基础还不坚实，发展速度还不够快；环境问题依然突出，节能减排压力巨大；社保体系还不健全，一些群众生活比较困难；影响社会稳定因素仍然存在，安全隐患没有彻底消除；推动科学发展能力有待提高，政务环境亟需优化；等等。这些问题，我们将高度重视，努力解决。

　　二、提振信心，科学谋划今年工作

　　2009年，是挑战与机遇并存、困难与希望相伴的一年，也是推进本届政府工作的关键一年。挑战给人压力——从全球看，金融危机已经演变为经济危机，美国、日本和欧元区经济陷入衰退，市场信心严重不足；从全国看，外需压迫内需，保增长的不确定性因素增多，下行风险加剧；从全市看，虚拟经济对实体经济的影响日益显现，房地产、有色、化工、陶瓷等行业面临严峻挑战，保持经济较快增长的压力加大。机遇催人奋进——其一，中国经济的基本面仍然较好，金融体系总体稳健，正在实施的积极财政政策和适度宽松货币政策，必将为经济增长提供坚强保障；其二，国家启动以交通、能源为重点的基础设施建设，株洲享有“两型社会”建设等六大国家层面的政策，产业结构与国家扩大内需的宏观政策契合度较高，必将给我们带来新的发展机遇和空间。

　　坚定信心迎挑战，抢抓机遇谋发展。我们将按照市委十届六次全会提出的“目标不变、速度不减、标准不降”的要求，突出在“保增长、强基础、调结构、促民生”上下大力，使2009年成为基础建设年、招商引资年、管理效益年。保增长——就是把保持经济平稳较快增长作为首要任务。既立足实际，又强调可能，牢牢把握发展主动权，“弯道超车”，“跳起来摘桃子”，以保增长来保就业，保收入，保统筹。强基础——就是把强化基础设施、加强基础工作、发展基础产业作为保增长的根本路径。继续抓住国家扩大内需的重大历史机遇，多谋长远，多争资金，多上项目。调结构——就是把调整产业结构、产品结构、企业组织结构、产业布局结构和消费结构作为保增长的主攻方向。推进产业高端化、产品终端化、企业规模化、布局集中化、消费多元化。促民生——就是把帮助群众解决利益问题作为保增长的出发点和落脚点。坚持量力而行，确保尽力而为，优化财政支出结构，提高公共服务水平。

　　政府工作的指导思想是：深入学习实践科学发展观，紧扣“两型社会”建设主题，解放思想，大胆创新，扩大内需，刺激消费，加快推进新型工业化、新型城市化和新农村建设，确保经济平稳较快增长，千方百计改善民生，切实加强自身建设，为实现“保二争一、科学跨越”战略目标而努力奋斗！

　　全市经济社会发展的预期目标是：GDP增长12.5%，跨过千亿元大关；财政收入、一般预算收入都增长13%；全社会固定资产投资增长30%以上，力争突破500亿元；社会消费品零售总额增长16%；城镇居民人均可支配收入、农民人均纯收入都增长10%；城镇登记失业率控制在4.5%以内；人口自然增长率控制在7.5‰以内；万元GDP能耗和主要污染物排放总量都下降5%以上；各项事业都有新的进步。同时，集中人力、财力和物力，突出抓好十大基础工程、十大产业项目、十件民生实事。

　　十大基础工程是：1、推动岳汝高速醴炎段开工，构筑区域高速圈。2、推动衡茶吉铁路加快建设和泉南高速茶陵段尽早启动，打造湘赣边界交通枢纽。3、开工建设迎宾大道，拓展城市东大门，带动云龙生态城建设。4、开工建设炎帝大道，畅通城市南大门，拓展河西新城。5、建成时代大道，完善城市北大门，推动长株潭三市融城。6、启动建设职教园，打造城市新名片。7、基本完成市中心医院主体工程，优化医疗资源布局。8、实质性启动湘江风光带建设，提高城市品位。9、改造炎帝广场，启动天台公园建设，方便市民休闲。10、建成6个污水处理厂，提前一年实现城镇污水处理设施全覆盖。

　　十大产业项目是：1、电力机车扩能过100亿元工程。2、唐人神1000万头生猪产业化跨100亿元工程。3、时代新能源、新材料过50亿元工程。4、加快建设攸县煤电一体化项目。5、加快建设北汽控股南方生产基地。6、完善长株潭国家航空航天高技术产业基地。7、开工建设中国釉下五彩陶瓷创意产业园。8、千金二期竣工投产。9、湖南创科1500吨多晶硅投产。10、株冶循环经济一期投产、二期开工。

　　十件民生实事是：1、做好就业再就业工作。稳定农村劳动力转移就业60万人；开展农村实用技术培训10万人次、农民职业技能培训2万人次；新增城镇就业3.9万人,其中失业人员再就业2.2万人；城镇“零就业家庭”保持动态为零。2、完善社保体系。启动农村居民养老保险试点；企业职工基本养老保险新增参保6万人，城镇居民医疗保险参保登记人数达到36万人。3、实施安居工程。新建农村安居房1000栋；新增廉租房8万平方米。4、发展教育事业。资助家庭贫困学生1.55万人；实施“阳光体育工程”，新建12个塑胶运动场。5、加强农产品流通管理。新建改造农贸市场20处；城区蔬菜农药残留检测率达到100%。6、改善农民出行条件。全面完成通乡公路改造，实现村村通公路；新建农村客运招呼站160个。7、加大社会救助力度。落实城乡低保标准，实现应保尽保。8、推广清洁能源。农村新建沼气池7000个；市区全面推进煤改气。9、维护饮水安全。帮助8.35万农村人口解决饮水不安全问题；力争启动城市第二水源建设。10、改善市区环境质量。新增城区绿地面积50万平方米；城市污水处理率、垃圾无害化处理率分别达到68%、100%。

　　三、突出重点，加快推进经济建设

　　保持全市经济平稳较快增长，重中之重是突出“两型社会”建设，加快新型工业化、新型城市化和社会主义新农村建设进程,推进改革开放。

　　坚持示范引路，推进“两型社会”建设。突出规划引领。进一步完善总体实施方案，编制完善核心区建设、土地利用、产业发展等总体规划和各项专业规划。以规划为引导，带动实施一批重大产业和基础设施项目。突出示范区建设。云龙示范区，重点是按照世界一流标准、代表未来城市发展方向的要求，实质启动云龙生态城可持续发展中英合作，高起点、高水平编制规划，做好红楠大道、林东路的前期工作，推进基础设施建设；加快启动清水塘循环经济示范区建设，提升改造传统产业，发展循环经济和环保产业，做足做活土地变性文章，开展首期污染土地综合治理，退出“两高一资”产能，加强“三废”治理，启动铜霞路的建设，完善路网结构。天易示范区，重点是依托高新区、欧洲工业园，发展高新技术产业，加快武广客运站片区和新马片区开发区建设。协调发展区，重点是发挥比较优势，改善基础设施，为丘陵山地发展现代农业积累经验。同时，积极抓好示范社区、示范学校、示范道路建设。突出双向融城。以高速公路、城际干道、一体化的城乡交通和湘江梯级开发为支撑，推动完善综合交通网络，加快北接长沙、西连湘潭步伐，实现三市资源共享，通讯同区号同费率。

　　坚持产业兴市，推进新型工业化。要突出工业主导地位，实施“5115”工程，打造千亿产业集群，构筑以“两型产业”为核心的产业体系，具体是四个“两手抓”：一手抓产业旗舰，坚定不移实行特殊政策、特殊奖励、特殊服务，推动“5115”企业产能和效益实现突破，全市规模工业增加值突破400亿元；一手抓中小企业，千方百计帮助解决土地和资金困难，培育壮大一批骨干民营企业和高科技、高效益企业，壮大非公经济。一手抓传统产业改造，着力政策创新和资源整合，推进节能降耗减排，全力打造交通装备制造、有色冶炼及深加工、服饰加工与贸易、健康食品等优势产业集群；一手抓高新技术产业发展，培育壮大新材料、生物医药和电动汽车、风力发电装备、多晶硅新能源等新兴产业，促进优秀科技成果向现实生产力转化，尽快形成一批自主知识产权和技术标准，造就一批知名企业和品牌。一手抓园区平台建设，支持高新区、循环经济工业园和陶瓷创意产业园做大做强，加快打造新能源、电子信息等特色园区，支持各县市发展工业园区和工业小区；一手抓服务环境建设，创新政务服务，优化发展环境，营造“企业围着市场转、政府围着企业转”的“洼地效应”。一手抓传统三产业的提升，改造升级商贸业，支持发展房地产业，培育新的经营业态和产业形态，加强旅游景点建设，打造炎帝陵、106国道、城郊休闲风光带“一点一线一带”旅游产业长廊；一手抓现代服务业的壮大，突出发展现代金融业、现代物流业、商业服务业、信息服务业等，提升对新型工业化的服务能力。

　　坚持城镇带动，推进新型城市化。按照以现代工业文明为特征的生态宜居城市的定位，坚持“内提外拓、统筹发展”的原则，积极推进“一提两改三化四创”，加快形成“一体三极、一圈三环”的城市发展新格局。一提，就是提升城市品位；两改，就是推进城市管理体制和城郊集体土地利用改革；三化，就是城乡一体化、管理精细化、运作市场化；四创，就是大力创建国家卫生城市、国家交通管理模范城市、国家环保模范城市和全国文明城市。“一体三极”，就是用“织补”理念提质改造旧城区，突出“一江两岸四港”，实质性建设湘江风光带，提高中心城区首位度；向北加快田心立交、长株高速、迎宾大道、时代大道和红楠大道、林东路的建设，推动云龙生态城的发展；向西抓好武广客运专线株洲站建设，开工珠江北路，延伸株洲大道，完善栗雨工业园路网，拓展河西新城；向南推进枫溪——伏波大道建设，壮大董家塅高科园，打造枫溪新城。同时，加快县城和小城镇提质扩容，重点建设炎陵霞阳生态古镇、茶陵云阳交通古镇、攸县梅州古镇和网岭能源新镇、醴陵均楚物流新镇，以及株洲县渌口、三门、雷打石三个休闲小镇等一批区位较好、实力较强、辐射能力较强的中心镇。“一圈三环”，就是建成衡炎高速、长株高速，加快建设炎睦高速，开工建设岳汝高速醴炎段、泉南高速茶陵段，构筑两小时通勤半径的区域高速圈；尽早建好湘江五桥，形成城市的内环；推进王家坪立交、东环北路等项目建设，完善城市的中环；争取醴潭高速南下，形成城市的外环。同时，更加注重功能配套，重点改善城市路网，实行公交优先，提升市政保障能力，完善公共服务设施；更加注重规范管理，重点是深入推进城市“美化、绿化、亮化、数字化”工程，积极开展“穿衣戴帽刷漆”、“拆围透绿栽树”，大力整治“灯饰广告店牌”，拆除主次干道临街建筑防盗窗网，建设智能防控体系，打造“平安株洲”；更加注重高效经营，推进市政公用事业改革，整合城市供水、排水、污水处理等事务，组建水务集团。

　　坚持统筹发展，推进新农村建设。认真贯彻党的十七届三中全会精神，以新农村建设统揽“三农”工作，更加重视农村基础设施，更加重视农业基础地位，更加重视农民增加收入。加强基础设施，努力实现“村庄美”。抓住国家加大农村基础设施投入的机遇，进一步抓好农村道路、农田水利、电力、通信和广播电视建设，改善农村生产和消费环境，完善文化、教育、科技、医疗等服务体系。重点抓好22个新农村建设示范点，真正起到示范作用。建成网朱公路，完成S313醴陵至株洲县、S321炎陵至睦村、S320茶陵至界化垄等3条干线公路扫尾工程；建成醴潭高速株洲东互通至云田、衡炎高速安攸互通至攸县连接线；启动S315龙下至攸县、S322资兴至牛岗排、醴潭高速芷线桥互通至渌口等3条干线公路建设；力争启动106国道炎陵至槽里段、省道S211株洲段建设。完成80座中小型水库病险治理，抓好桐坝水电站、洮水水库、酒埠江灌区等大型水利设施建设，开展洣水流域综合治理。发展现代农业，努力实现“村民富”。稳定种植面积，提高综合生产能力，确保粮食稳产增收。科学布局、体现特色，抓好106国道沿线和城郊休闲农业两大产业带建设，建成一批无公害、绿色食品生产基地，发展壮大一批农产品加工龙头企业。抓好培训，就地转移，大力发展劳动密集型产业，让务工农民“离土不离乡、致富不离村”。支持发展专业合作经济组织，健全社会化服务体系，提高农业市场化程度和抗风险能力。促进乡风文明，努力实现“村风好”。加强社区建设，强化基层组织，落实村民自治，扩大基层民主。加强法制教育，繁荣文化事业，提高农民素质，培育现代农村新风尚。

　　坚持先行先试，推进改革开放。改革方面，重点是按照国家批复的长株潭城市群综改方案要求，推进重点领域和关键环节改革，率先形成有利于资源节约、环境友好的新机制，率先形成城市发展的新模式，率先积累传统工业成功转型的新经验。以户籍制度改革为突破口，启动城市四区农民转城工作，加快城中村改造，逐步实现农民变市民、村庄变社区、土地变国有。继续深化行政审批制度改革，推进政府信息公开和网上办公。基本完成已启动的工业企业、集体企业、建设和商贸企业改革。大力深化乡镇机构改革，深入推进城乡建设用地增减挂钩试点。出台农村土地和集体林权流转实施办法，推动流而有向、流而有序、流而有效。落实成品油价格改革举措，取消二级公路收费。开放方面，重点是做好产业承接工作，着力解决土地、资金、能源等瓶颈制约，加强与央企对接，努力推动与长三角、珠三角等地区在项目、技术、金融等方面的合作。进一步增强招商实效，树立“大招商、招大商”的理念，完善招商引资奖励办法，探索公司化招商模式，大力引进战略投资者，力争优势产业招商、重大项目招商取得突破。进一步优化进出口结构，促进加工贸易转型升级，扩大高技术和高附加值产品的出口。进一步深化对外合作，支持企业“走出去”，带动产品和劳务输出。积极发展与国内外城市的友好关系。进一步完善创业平台，健全服务体系，营造良好环境，鼓励、引导和支持一切有创业愿望和能力的企业、个人创新创业。

　　四、以人为本，全面抓好社会建设

　　坚持发展成果由人民共享，切实保障和改善民生，全面加强社会建设，不断增强人民群众的幸福感，致力打造“民本”政府。

　　推进科教先导。健全科技创新投入增长机制和成果转化机制，建立和完善以企业为主体、市场为导向、产学研资相结合的区域创新体系；依托湖南工业大学、株洲高新区等创新平台，实施重大科技专项，推动轨道交通、电动汽车、风电装备三大战略联盟取得实质性进展，培养创新型人才，培育创新型企业，打造创新型城市；重视基础研究，抓好科普工作，鼓励自主创新，提高城市软实力。强化教育的基础性、战略性、先导性地位，切实抓好各级各类教育，提高人民满意度，推进教育强市进程；加强学校品牌和教师队伍建设，着力培育一批名校、名师、名校长。

　　着力文化提升。倡导核心价值。坚持社会主义取向，着力培育和铸就株洲精神，增强全市人民的归属感、向心力。夯实文化载体。加快推进炎帝文化艺术中心建设，新建改造市规划馆、图书馆、博物馆、群众艺术馆、河东体育馆，大力建设乡镇综合文化站和村文化室，为城乡居民文化休闲创造条件。打造文化品牌。围绕群众文化、炎帝文化、陶瓷文化和服饰文化等地方特色文化，挖掘文化内涵，繁荣文化创作，开发文化精品。活跃文化生活。创新企业文化、社区文化、军营文化和广场文化等文化形式，抓好建国60周年庆祝活动以及读书月、炎帝节、服饰节、周周乐和“五下乡”等，引导健康、向上的社会风尚和生活方式。培育文化产业。大力发展文化创意、文化旅游、文化休闲等产业，推动产业优化升级。

　　发展各项事业。大力开展爱国卫生运动，着力完善疾病预防体系和公共卫生体系。整合医疗卫生资源，推进社区卫生服务中心、乡镇卫生院、村卫生室和市中心医院、中医院、人民医院、伤科医院、妇幼保健院建设。巩固广播电视“村村通”成果，全面建成广电中心，打造全省一流的广播电视阵地。广泛开展群众体育运动，努力办好市第十一届运动会。深入开展“扫黄打非”，繁荣新闻出版事业。抓好民族宗教、外事侨务、地方志、档案等各项工作。

　　提高保障水平。突出民生之本，把促进就业摆在更加重要的位置，加强政府引导，完善就业服务和援助体系，特别注重解决返乡民工和失地农民的就业困难。做实民生之依，进一步强化社会保险扩面措施，加快实现城镇从业人员的养老、医疗和工伤、失业、生育保险全覆盖。强化民生之助，加大项目、产业、智力、技能扶贫力度，帮助更多困难群众脱贫致富。保障残疾人合法权益，提高残疾人保障水平。落实家电下乡政策，提高农村电器普及率。大力发展妇女儿童、社会福利、慈善、法律援助等社会公益事业，积极构建新型社会福利体系。

　　建设生态文明。坚持可持续发展战略，切实转变发展方式，努力实现人口增长、资源利用和环境保护的协调统一。认真落实计划生育工作机制，稳定低生育水平，提高出生人口素质。坚决执行“环评”和“三同时”制度，严格落实“五个一律不批”，切实把好新建项目环保审批关。深入开展“蓝天碧水净土”行动，全面推进“一江两岸四港”整治，着力把湘江打造为“东方莱茵河”，把四港打造成城市亮丽的风景线，力争城区空气良好在330天以上，市区饮用水源水质达标率稳定在98%以上。加强农村污染防治，严控重污染企业和落后产能向农村转移，妥善解决规模化养殖的污染问题。继续抓好植树造林，完成10万亩工程造林任务。注重节地、节水、节材，加强耕地保护和土地开发整理，集约利用能源和矿产资源。

　　加强社会管理。健全社会治安防控体系，强化社会治安综合治理，依法严厉打击各类违法犯罪，切实增强人民群众安全感。严格落实安全生产责任制，加强对重点行业、重点领域的监管整治，防止重特大事故发生。继续抓好“法治株洲”建设，加强和改进信访工作，坚持领导干部开门接访、带案下访，妥善排查化解矛盾纠纷。完善应急体系，提高应对突发事件能力。加强产品质量安全监管，完善社会信用和担保体系，严厉打击制假售假、欺行霸市、商业欺诈、非法金融、偷税漏税等不法活动。大力推进政务、厂务、村务公开，加强城市社区管理，扩大农村社区试点范围。同时，加强国防教育，做好人民防空和民兵预备役工作，力争夺取全国双拥模范城“五连冠”。

　　五、首重执行，努力加强自身建设

　　历史的使命感和现实的责任感，让我们清醒认识到，必须加强政府建设，提升执行力，才能推进全市经济社会再上新台阶。

　　加强民主法制。始终坚持党的领导，自觉接受人民代表大会及其常委会的法律监督和工作监督、人民政协的民主监督、新闻舆论和社会公众监督，不断改进政府工作。进一步完善政府规章，严格按照法定权限和程序行使权力，提高依法管理社会事务的能力。强化行政执法监督，规范行政执法裁量权。充分重视各民主党派、工商联、无党派人士和社会各界的参政议政，支持工会、共青团、妇联等人民团体发挥桥梁和纽带作用，让人民群众更广泛地参与社会管理和公共事务。

　　完善体制机制。完善科学民主的决策机制。坚持解放思想，实事求是，以宽广视野把握大势，以战略思维谋划全局，认真研究经济社会发展的宏观走势和微观变化，及时发现和把握倾向性问题，突出决策的前瞻性，提高决策的准确性，避免决策的盲目性；落实重大事项集体决策、专家咨询、社会公示与听证等制度，保证决策的科学化、民主化。完善精简效能的执行机制。善于运用现代手段管理政府事务，提高行政效率；进一步精简行政机构，优化行政流程，着力解决机构重叠、职能交叉、责任不清等问题。完善奖罚分明的激励机制。建立科学的绩效评价体系，加大政绩考核力度；严格兑现绩效考核结果，适当拉开分配档次，着力营造“能上也能下”、“能进也能出”的公务员管理和激励机制。完善权责统一的问责机制。进一步规范职责范围和运行程序，制定详细的效能目标，切实搞好目标分解；按照“不负责，就问责”的原则，做好行政行为评议，推行电子监察，严格责任追究，确保权责明晰、落实得力。

　　转变工作作风。牢固树立以重点带全局的思想。善于“弹钢琴”，抓住主要矛盾和矛盾的主要方面，分清轻重缓急，以重点突破带动各项工作落实。牢固树立全市一盘棋的思想。讲大局、讲服从，保重点、保民生，增强整体作战能力。牢固树立过紧日子的思想。继续发扬艰苦奋斗优良传统，坚决杜绝铺张浪费之风，少花钱多办事，不花钱也办成事，努力降低行政成本，提高管理效益。牢固树立清正廉洁的思想。全面落实廉政建设责任制，加大对公共支出、重点工程、专项资金的监察和审计力度，加强对公务员的教育、管理和监督，努力塑造为民、务实、清廉的政府形象。

　　各位代表！新的目标令人鼓舞，新的征程任重道远。让我们在市委的坚强领导下，围绕“保二争一、科学跨越”战略目标，以高昂的斗志、饱满的热情、超常的举措、务实的作风，同心同德，创新进取，为实现全市经济社会又好又快发展而努力奋斗！
